# HoMEcare aRm rehabiLItatioN (MERLIN): preliminary evidence of long term effects of telerehabilitation using an unactuated training device on upper limb function after stroke

**DOI:** 10.1186/s12984-021-00934-z

**Published:** 2021-09-19

**Authors:** Samantha G. Rozevink, Corry K. van der Sluis, Juha M. Hijmans

**Affiliations:** University of Groningen, University Medical Center Groningen, Department of Rehabilitation Medicine, PO Box 30001, 9700 RB Groningen, The Netherlands

**Keywords:** Stroke, Rehabilitation, Telerehabilitation, Home training, Upper limb, Hand, Training device, Task specific, Serious games

## Abstract

**Background:**

While short term effects on upper limb function of stroke patients after training with robotic devices have been studied extensively, long term effects are often not addressed. HoMEcare aRm rehabiLItatioN (MERLIN) is a combination of an unactuated training device using serious games and a telerehabilitation platform in the patient’s home situation. Short term effects showed that upper limb function improved after training with MERLIN. The aim was to determine long term effects on upper limb function and quality of life.

**Methods:**

Six months after cessation of the 6 week MERLIN training program, the upper limb function and quality of life of 11 chronic stroke patients were assessed. Upper limb function was measured using the Wolf Motor Function Test (WMFT), Action Research Arm Test (ARAT) and Fugl-Meyer Assessment-Upper Extremity (FMA-UE). EuroQoL-5D (EQ-5D) was used to measure quality of life.

**Results:**

The WMFT, ARAT and EQ-5D did not show significant differences 6 months after the training period compared to directly after training. At 6 months follow-up, FMA-UE results were significantly better than at baseline. Time plots showed a decreasing trend in all tests.

**Conclusion:**

Training effects were still present at 6 months follow-up, since arm function seemed similar to directly after training and FMA-UE results were better than at baseline. However, because of the decreasing trend shown in all tests, it is questionable if improvements will be maintained longer than 6 months. Due to the sample size and study design, results should be interpreted with caution.

*Trial registration* This study is registered at the Netherlands Trial Register (NL7535). Registered 18-02-2019, https://www.trialregister.nl/trial/7535

## Introduction

The majority of people who suffered a stroke have persistent problems with using the arm or hand in daily life, with estimates between 62 and 88% [1, 2]. After a severe paresis, only 7–18% regains full function of the upper limb [1, 3]. Actual numbers might be less drastic since these studies are rather dated and specific groups of severely affected stroke patients were investigated. Nevertheless, the latest insights show that improvement of the upper limb function is possible even 1 year after stroke onset [4, 5]. More training possibilities have become available for patients in the chronic phase of stroke. Patients with a better upper limb function may be more independent and less reliant on their caregivers, which is important for a stroke survivor [6]. An improved arm function could also lead to more use of the arm in daily life which in turn is beneficial for further recovery. Additionally, independence of the patient may unburden the health care system due to a lower need for care and adaptations at home or a reduction in the need for long term therapy. More frequent, intensive or longer training programs have been positively correlated with improvement in upper limb function [7]. Many patients would like to receive more training than they are currently offered, unfortunately this is not possible in all instances due to limited healthcare resources [6]. The search for alternative ways to provide therapy is on-going.

A promising solution is to use (robotic) devices to train the upper limb using serious games. Several reviews revealed that robotic training is safe and can provide more intensive training than in standard care situations, especially in the chronic phase [8–10]. Robotic training devices seem to improve the upper limb function directly after training [11, 12]. While these are only short term effects, equally important is to know whether training with these devices results in long lasting improvements. In only one review the follow-up after training was described, and the authors concluded that there was no significant difference between a robotic training group and the conventional group when matched for training intensity [8]. This conclusion was based on a meta-analysis using data from only three papers in which two investigated a robotic device and one a non-robotic device [8, 13–15]. Evidently, insufficient information is yet available on the long term effects of training with (non)robotic devices.

**Fig. 1:**
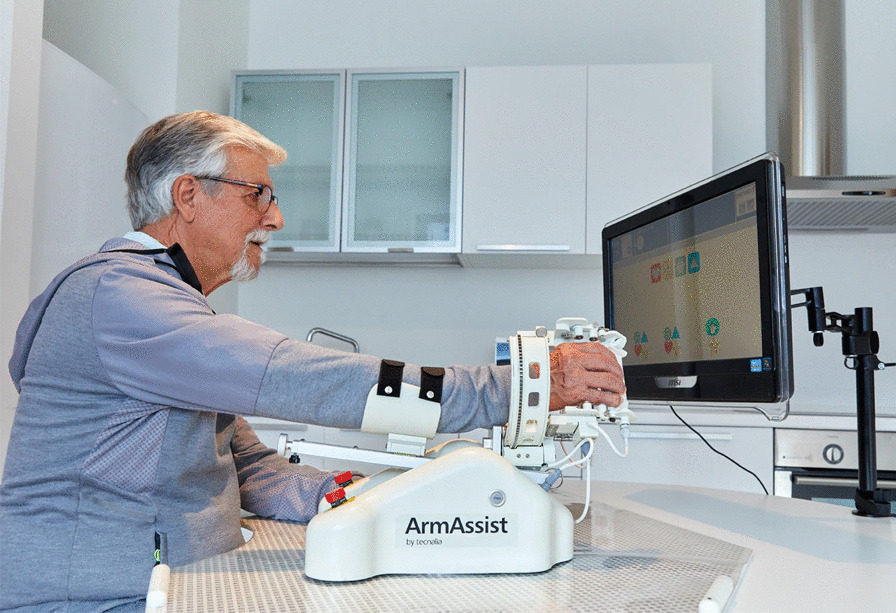
hoMEcare aRm rehabiLItatioN system for training the upper limb function at home

We investigated the long term effect of hoMEcare aRm rehabiLItatioN (MERLIN), an unactuated (non-robotic) training device combined with a telecare platform to train the arm and hand at home (see Fig. 1). The short term results of MERLIN showed that moderately affected patients in the chronic phase of stroke were able to achieve a statistically significant as well as a clinically relevant improvement in upper limb function [16]. The improvements were retained at least 6 weeks after termination of the training. We expected patients to use the affected arm more in daily life after the intervention due to an improved arm function. Some evidence suggests that after intense therapy, patients are able to use the hand more during different activities with effects lasting up to 1 year [17, 18]. Therefore we hypothesized that upper limb function would improve between cessation of the intervention and 6 months after the intervention. The aim of this study was to assess upper limb function and quality of life in patients in the chronic phase after stroke, who trained with MERLIN at home 6 months prior to the assessment. First we will discuss the difference between our findings directly after the training and the findings at 6 months follow-up. Thereafter, we compare the 6 month follow-up data to the data of all previous measurements.

## Methods

### Subjects

Patients in the chronic phase after a single stroke (> 6 months and < 3 years) were eligible. Patients had to be able to extend their fingers, but Fugl-Meyer Assessment-Upper Extremity (FMA-UE) score had to be lower than 50. Extended inclusion and exclusion criteria were published elsewhere [16]. The study was approved by the local Medical Ethics Committee of the University Medical Center Groningen (UMCG) (METc 2019/189). All patients provided written informed consent before participating.

### Design

**Fig. 2:**
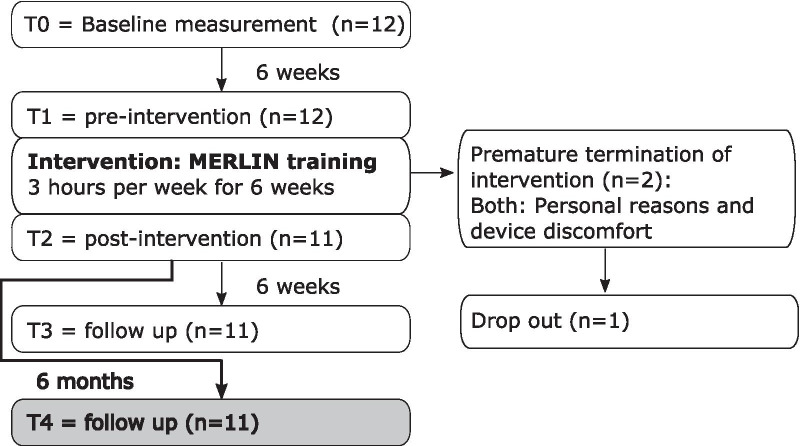
Flow diagram of the study. n = number of patients. Two patients terminated the intervention prematurely, of whom one still participated in remaining measurements T2, T3 and T4. Grey shade = measurement described in the current paper. All other measurements (white boxes) have been described in [16]

Participants were measured repeatedly over the course of 9 months (see Fig. 2). Data gathered for this paper concerned measurements at 6 months follow-up which were added to the existing dataset. The long term measurement was not part of the original MERLIN grant proposal and was therefore considered as a separate study. Patients who participated in the previous part of the trial were invited via a phone call for the final follow-up measurement. During the intervention period, one patient dropped out after 3 weeks and was unable to complete the post-intervention measurement (T2, see Fig. 2). Due to the short training time and the patient’s fragility, she was excluded for further measurements. Another participant stopped training after 5 weeks, but was able and willing to continue the follow-up measurements. All T4 measurements of the eleven patients took place between May and November 2020.

### Intervention

The details of the intervention were described previously [16], but main characteristics will be mentioned here as well. Participants received the MERLIN system at home, consisting of the ArmAssist device, laptop and placemat (the surface on which the ArmAssist was used). The ArmAssist is a low-cost unactuated device, which behaves similarly to a computer mouse. The ArmAssist was used to play serious games on a computer. In addition, it allowed the user to train several movements with the arm, wrist and hand. The laptop contained the telerehabilitation software and the serious games. Via the telerehabilitation platform the therapist was able to set the training games, monitor therapy compliance through the number of played games and send and receive messages from the patient.

The therapy provided was task specific of nature; intensive and repetitive, targets were presented randomly in the visual work space, visual feedback and positive reinforcement was provided [19]. A calibration procedure was performed every 2 weeks to adjust the system to the participant’s capabilities to maintain a challenging environment. In the intervention period, participants were instructed to train with MERLIN 3 hours per week for 6 weeks, aiming at a total training time of at least 18 h [2]. Patients were able to determine their own training time and duration, although a minimum of 3 h per week was requested.

From T0 to T3, patients did not follow any other functional arm or hand therapy. However, between T3 and T4 they were allowed to resume their previous therapy or start new therapies.

### Outcomes

Outcomes were based on the International classification of Functioning, Disability and Health to classify the patient’s health state, based on body functioning, activities and participation [20]. Main outcomes were three upper limb function tests: FMA-UE from the body function domain, Wolf Motor Function Test (WMFT) and Action Research Arm Test (ARAT) from the activities domain. These three common tests are widely used in stroke research and will be briefly addressed below [21]. To cover the participation domain, the quality of life questionnaire EuroQoL-5D was used.

The primary outcome was the WMFT, which consists of two scores: the time score per item and the Functional Ability Scale (FAS) [22]. Per item the maximum score is 120 s, with lower completion time indicating better performance. The FAS is a score between 0 and 5 to score the patient’s movement on dexterity, fluency and speed, with a maximum of 75 points. The FMA-UE scores the upper limb function in four categories: shoulder, wrist, hand and speed/coordination [23]. Every movement is scored between 0 and 2, with 66 being the maximum score to indicate normal motor functioning. The ARAT contains the subscales grasp, grip, pinch and gross arm movement. The movements performed in the ARAT are scored on a four point scale (0–3) with a maximum of 57 points [24].

In order to evaluate a long term improvement in the arm function of chronic stroke patients, the minimal clinically important difference (MCID) was taken into account for every arm function test. The MCID reflects scores that are considered clinically relevant to the patient. The MCID is 3–6 points, 6–8 points and 5.7 points for the WMFT, FMA-UE and ARAT, respectively [25–27].

The quality of life was measured using the EuroQoL-5D-5L, containing a visual analogue scale (VAS) and a questionnaire of five questions which can be combined to calculate a health state [28]. The MCID for the EQ-5D is 0.1 for the health state and 8.6 for the VAS [28].

### Measurements

Measurements and training took place at the participant’s home. An independent researcher was trained by an occupational therapist to perform the arm function tests. Blinding of the independent researcher was not possible due to the design of the study.

### Statistical analyses

REDCap (Research Electronic Data Capture) was used to manage the data, servers were hosted at UMCG [29, 30]. REDCap is a secure, web-based software platform designed to support data capture for research. IBM SPSS Statistics (version 23) was used to perform statistical analyses. Data from the previous trial (T0 (baseline, 6 weeks before intervention), T1 (pre intervention), T2 (post intervention) and T3 (6 weeks retention)) were used in addition to the data gathered in this study. Data was tested for normality using Shapiro–Wilk test and z-scores for skewness and kurtosis. For parametric testing, a repeated measures ANOVA with a Bonferroni correction for multiple measurements was used (alpha = 0.05). Effect sizes were calculated using partial eta squared (η_p_^2^). Non-parametric Friedman tests were used if normality was not met. Pearson correlation coefficients were calculated to investigate possible confounding effects of training time on upper limb function at follow-up. Because of the small sample size, an analysis on the observed trends was added.

## Results

Patients were allowed to resume any type of therapy if desired (Table 1). One patient suffered a second stroke, but no clinical rehabilitation was necessary after hospitalization.

**Table 1:** Patient characteristics at baseline and consumed additional therapy between six weeks (T3) and 6 months (T4) after cessation of the intervention (T2) (N = 11)

	Mean ± standard deviation
Age (years)	66.0 ± 8.4
Time after stroke (months)	22.5 ± 9.7

	N
Sex (male/female)	8/3
Type of stroke (haemorrhagic/ischemic)	1/10
Dominant side (left/right)	0/11
Affected side (left/right)	7/4
Consumed additional therapy between T3–T4	
Physiotherapy (arm and leg)	3
Fitness training	2
Yoga	1
Neurofeedback therapy	1
Dry needling	1
None	2

Due to the COVID pandemic, the 6 month follow-up measurement of two patients had to be performed via an online video call. The measurement equipment was delivered at the patient’s house and via the online video call the researcher instructed the patient during the measurements. Two assessors were online present to assess the scores and to reach consensus on the final scores. The T4 measurements of the other patients were performed physically, taking into account the necessary health precautions.

**Fig. 3:**
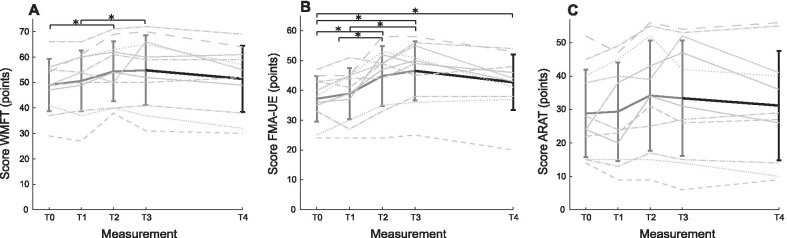
Mean scores on the Wolf Motor Function Test (**A**), Fugl-Meyer Assessment – Upper Extremity (**B**) and Action Research Arm Test (**C**). *ARAT* Action Research Arm Test; *FMA-UE* Fugl-Meyer Assessment – Upper Extremity; *WMFT* Wolf Motor Function Test. *T0* baseline; *T1* pre-intervention; *T2* post-intervention; *T3* follow-up 6 weeks; *T4* follow-up 6 months. Thick grey lines represent previous measurements; thick black lines represent the additional new data; light grey striped/dotted lines represent individual data. Vertical bars represent standard deviation. *Significant difference, N = 11

In this section we will present the findings related to the T4 measurements (see Fig. 3). Other findings shown in Fig. 3 can be found in Appendix 1 and have been discussed previously [16].

### Statistical effects

Data was normally distributed and thus a parametric ANOVA was used for the analysis. For the primary outcome, the WMFT FAS, a significant main effect was found (F(4,40) = 6.94, p < 0.001, η_p_^2^ = 0.4). Post hoc analysis showed that this difference could not be attributed to a difference between T4 and other measurements.

Regarding the secondary outcomes, significant main effects for the FMA-UE (F(4,40) = 15.02, p < 0.001, η_p_^2^ = 0.6) and ARAT (F(4,40) = 3.67, p = 0.012, η_p_^2^ = 0.27) were found. FMA-UE post hoc analysis showed a significant improvement of 5.5 points between T0 and T4 (p = 0.016). Post hoc analysis of ARAT did not yield any significant differences. No significant differences were found between the measurements in quality of life on the EQ-5D questionnaire scores (F(4,40) = 1.92, p = 0.126) or on the VAS (F(4,40) = 1.53, p = 0.211).

The mean training time was 16.4 ± 7.7 h. The training time did not seem to influence the results at follow-up. Pearson correlation calculations for training time and the differences in arm performance between T2 and T4 resulted in low and non-significant correlation coefficients for FMA-UE (r = − 0.161, p = 0.637), WMFT (r = 0.075, p = 0.826) and ARAT (r = − 0.122, p = 0.721).

### Observed trends

An observed trend in all performance tests was that patients’ upper limb function seemed to decrease slightly from cessation of intervention to 6 months post intervention. However, this decrease was not statistically significant. Visual inspection on an individual level showed that patients either increased or decreased a few points on the arm function tests. A lot of variation in arm function scores could be observed between the patients (see Fig. 3).

## Discussion

In this short report we present the results of a follow-up study 6 months after cessation of a home rehabilitation program using an unactuated training device. The WMFT and ARAT did not show significant differences 6 months after the training period compared to previous measurements. This could indicate that arm function improvement, established after 6 weeks of MERLIN training, remained stable on the ICF activity level. Due to the small sample size, results can be seen as preliminary evidence and should be investigated further. A significant increase was found between T0 and T4 for the FMA-UE, which showed that the arm function was significantly better 6 months after the training compared to the baseline measurement. Visual inspection of the data however showed that over time a decreasing trend was observed in all tests. It is therefore questionable if improvements will be maintained after a longer follow-up time than the 6 months we took into account.

Contrary to our hypothesis, arm function did not increase over time. We expected that arm function would continue to improve after the intervention as a result of using the arm more during activities, based on data from constraint induced movement therapy (CIMT) [17, 18]. Kwakkel et al. showed that after CIMT, patients increased their arm-hand activities, but not basic activities of daily living at long term follow up [18]. Waddell et al. showed that despite intensive training, patients did not change their use of the affected upper limb in daily life [31]. It seems that improved arm function does not always transfers to activities. We measured on all three ICF levels. The significant improvement compared to baseline in the FMA-UE scores suggests that patients improved on the ICF body function domain. WMFT, ARAT and EQ-5D did not improve compared to baseline, suggesting that the improvement in body functions did not transfer to improvements in activity or participation level. Although clinically relevant improvements of the upper limb above the MCID were observed directly after training [16], these changes were insufficient to result in a relevant change on all ICF levels. Apparently the relation between MCID and performance on ICF levels needs further investigation. A non-significant positive correlation between training time and differences in arm performance between T2 and T4 was found for the WMFT. This positive correlation could indicate that patients who trained more hours deteriorated less over time. However for the FMA-UE and ARAT, the non-significant correlation was negative, which may reflect that patients who trained more hours, declined more in arm function. Measurements errors should be taken into account and the small sample size makes it difficult to draw conclusions regarding these correlations.

An explanation for the observed trend that arm function seems to be returning to baseline could be that, over time, patients stop using the affected arm/hand in daily life. They relapse into old behaviour of relative disuse of the affected arm in favour of the non-affected arm. Another reason could be that the transfer to daily activities of what is learned using a device is more difficult than when traditional training schemes are applied. Generalizability of training is known to be difficult in traditional rehabilitation [32]. How effects of device training can be generalized, has not been investigated yet.

The few studies that investigated long term results (6 months or longer) after the use of an assistive or robotic device reported long lasting improvement of the upper limb function. Housman et al. trained 14 chronic stroke patients with the non-robotic Therapy Wilmington Robotic Exoskeleton (T-WREX) [14]. After 6 months the upper limb function was still significantly better compared to baseline. Studies investigating the long term effect on upper limb function of robotic devices such as mirror image movement enabler (MIME) and MIT-Manus yielded similar results [15, 33]. Although the improvement in arm function was maintained over time, the absolute improvement in all three studies did not exceed the MCID on the FMA-UE, nor the minimal detectable change (MDC: 5.2 points [26, 34]). The MDC is a measure to account for “noise” in the scoring, improvement below the MDC could therefore also be attributed to measurement errors. The FMA-UE difference between T0 and T4 in the current study was 5.5 points, and thus larger than the MDC. The improvements from our and aforementioned studies are small and around the MDC, which means that outcomes may have been affected by measurement errors and therefore should be interpreted with caution. An explanation why our study was able to achieve significant improvement on the FMA-UE, above the noise level, could be the addition of the hand module. The T-WREX, MIME and MIT-Manus only train shoulder and elbow movements. The addition of wrist and finger movements makes MERLIN more versatile. The trends in our study showed that results on the shoulder and wrist part of the FMA-UE declined from T2 to T4, however the results obtained from the hand section seemed to remain constant. This may explain why improvement in FMA-UE score was larger in the MERLIN study than in the literature.

Breaking the habit of learned non-use is known to be difficult [35], which may also have been the case in our study. Similar effects have been shown in other therapy forms such as CIMT [18]. Motor function improvements remained stable compared to the start of the training, however effect size decreased over time [18]. One of the key points to maintain the improvement seems to be the dose of the training. Ward et al. showed that an intensive training of 90 h in 3 weeks resulted in long-lasting improvements of 11 points on the FMA-UE after 6 months [36]. Although this study was not executed at the patients’ home and not solely performed using training devices, it may open perspectives for intensive home training programs.

To maintain arm function levels after training, it could be interesting to investigate the effect of a repeated intervention program. Such an attempt has previously been made by Sale and colleagues: patients trained twice using the ReoGo robot device for 1 month, with 3 months in between [37]. At follow-up, 6 months after the second period of training, improvements in the Box and Block test and the Frenchay Arm Test appeared to be retained. FMA-UE scores returned to baseline during the 6 month follow-up. Apparently, this protocol is contradictory to our outcomes, since the ICF activity level was maintained while the body function was not. Future studies could investigate the effect of repeated periods of exercise more closely for a longer follow-up duration. Questions regarding the length of the exercise period and the time between exercise periods are of interest. Perhaps training programs of either high dose sessions from time to time or a continuous low dose of exercise are necessary to maintain the improved arm function.

Some limitations of the study have to be mentioned. Firstly, COVID-19 may have had an effect on the long term retention of the arm function. Due to COVID-19 restrictions, patients may have been less challenged in engaging activities in daily life or in receiving additional therapies. Secondly, the small sample size of this study is a limitation. Due to limited availability of the devices and time restrictions of the initial project, only 12 patients were able to participate of whom 11 completed all measurements. Another limitation is the pre-post-test design of the study. A randomized controlled trial may provide stronger evidence about the long term effects. Additionally, patients were able to resume other therapies after the T3 measurement. The therapies that were followed by the patients did not seem to have affected our outcomes since they were provided in a low dose (max twice a week) and none of the therapies focussed solely on the arm/hand function. Lastly, to conclude whether the arm function remained stable in the retention period, equivalence testing should have been used. However, this is not possible for within subject multiple measurements in a limited sample size. A large sample size is necessary to perform such tests. Therefore, we cannot conclude that the improved score after 6 weeks training is similar to the score after 6 months follow-up. We can only conclude that arm function did not significantly change during follow-up.

## Conclusion

None of the arm function tests showed significant or clinical relevant differences in the period directly after cessation of the MERLIN intervention and 6 months later, indicating that the improved arm function did not change after cessation of the training. A significant improvement was found in the FMA-UE scores, indicating an improved arm function 6 months after training compared to baseline. Visual inspection of test results showed however a decreasing trend for all arm function tests between post-intervention and 6 months follow-up. It is questionable if the improvement in arm function found after the intervention will be maintained after a longer follow-up period. No differences were observed in quality of life. In terms of ICF, MERLIN seemed to result in long term improvements in the body functions domain, but not yet on activity or participation level. Future studies should investigate the effect of different training programs including short high dose or continuous low dose treatments. Finally, a RCT is necessary to strengthen our results.

## Data Availability

The dataset supporting the conclusions of this article are available via DataverseNL: 10.34894/MBABRA.

## Appendix

Results of all outcome measures for all measurement times during the MERLIN study (N = 11)

**Table:**

Outcome measures	Mean ± SD					Post hoc analysis p-value
	T0	T1	T2	T3	T4	
WMFT FAS	49.0 ± 10.3	50.6 ± 11.9	54.4 ± 11.8	54.8 ± 13.8	51.5 ± 13.0	T0–T2 = 0.025 T1–T3 = 0.048
WMFT Time	1.9 ± 0.4	2.2 ± 0.6	2.0 ± 0.5	2.0 ± 0.4	1.7 ± 0.3	
FMA-UE Total	37.2 ± 7.6	38.9 ± 8.6	44.8 ± 10.1	46.5 ± 9.9	42.7 ± 9.3	T0–T2 = 0.020 T0–T3 = 0.001 T0–T4 = 0.016 T1–T2 = 0.042 T1–T3 = 0.001
FMA-UE Shoulder	21.8 ± 3.9	22.5 ± 4.0	25.6 ± 5.4	26.7 ± 4.9	24.7 ± 4.5	T0–T3 = 0.012 T0–T4 = 0.029 T1–T3 < 0.001
FMA-UE Wrist	6.0 ± 2.8	5.4 ± 2.0	5.8 ± 2.4	6.5 ± 2.4	5.3 ± 2.1	
FMA-UE Hand	6.5 ± 3.1	7.5 ± 3.8	8.9 ± 4.0	8.7 ± 4.1	8.8 ± 3.7	T0–T2 = 0.035
FMA-UE Coordination/ speed	2.8 ± 1.3	3.5 ± 1.3	4.5 ± 1.2	4.6 ± 1.2	3.9 ± 1.3	T0–T2 = 0.007 T0–T3 = 0.007
ARAT	28.8 ± 13.1	29.4 ± 14.7	34.2 ± 16.5	33.4 ± 17.3	31.2 ± 16.3	
EQ-5D health state	72.3 ± 18.4	68.3 ± 16.9	78.8 ± 16.7	72.5 ± 15.9	75.5 ± 15.7	
EQ-5D VAS	0.69 ± 0.12	0.73 ± 0.14	0.75 ± 0.08	0.74 ± 0.10	0.78 ± 0.08	

Repeated measures ANOVA with post hoc analysis. Only significant differences are shown, significant difference = p < 0.05.

*ARAT* Action Research Arm Test; *EQ-5D* EurQoL-5 Dimensions; *FAS* Functional Ability Scale; *FMA-UE* Fugl-Meyer Assessment – Upper Extremity; *SD* standard deviation; *VAS* Visual Analogue Scale; *WMFT* Wolf Motor Function Test. *T0* baseline; *T1* pre-intervention; *T2* post-intervention; *T3* follow-up 6 weeks; *T4* follow-up 6 months

## Abbreviations

ARAT: Action research arm test
CIMT: Constraint induced movement therapy
EQ-5D: Euro-Quality of Life-5 Dimensions
FAS: Functional ability scale
FMA-UE: Fugl Meyer assessment-upper extremity
ICF: International classification of functioning, disability and health
MCID: Minimal clinically important difference
MDC: Minimal detectable change
MERLIN: HoMEcare aRm rehabiLItatioN
MIME: Mirror image movement enabler
SD: Standard deviation
T-WREX: Therapy Wilmington robotic exoskeleton
UMCG: University Medical Center Groningen
VAS: Visual analogue scale
WMFT: Wolf motor function test
